# An Insight into the Biology of the Rare and Peculiar Moss *Pterygoneurum sibiricum* (Pottiaceae): A Conservation Physiology Approach

**DOI:** 10.3390/plants12061359

**Published:** 2023-03-17

**Authors:** Bojana Z. Jadranin, Marija V. Ćosić, Djordje P. Božović, Milorad M. Vujičić, Michael S. Ignatov, Elena A. Ignatova, Aneta D. Sabovljević, Marko S. Sabovljević

**Affiliations:** 1 Institute of Botany and Botanical Garden Jevremovac, Faculty of Biology, University of Belgrade, Takovska 43, 11000 Belgrade, Serbia; 2 Faculty of Biology, Lomonosov Moscow State University, Leninskie Gory Str. 1-12, 119234 Moscow, Russia; 3 Tsitsin Main Botanical Garden, Russian Academy of Sciences, Botanicheskaya Str. 4, 127276 Moscow, Russia; 4 Department of Botany, Institute of Biology and Ecology, Faculty of Science, Pavol Jozef Šafárik University in Košice, Mánesova 23, 040 01 Košice, Slovakia

**Keywords:** bryophyte, *Pterygoneurum*, protection, eco-physiology, functional features, in vitro

## Abstract

The biological features of the recently described peculiar and rare pottioid moss species *Pterygoneurum sibiricum* have been studied. A conservation physiology approach through in vitro axenic establishment and laboratory-controlled tests was applied to learn more about its development, physiology, and ecology. Additionally, ex situ collection for this species was established, and a micropropagation methodology was developed. The results obtained clearly document its reaction to salt stress in contrast to its sibling bryo-halophyte species *P. kozlovii*. The reaction to exogenously applied plant growth regulators, auxin and cytokinin, can be used in the different moss propagation phases of this species or for target structure production and development. Inference to the poorly known ecology of this species should also help in recent species records, and thus improve knowledge about its distribution and conservation.

## 1. Introduction

The moss *Pterygoneurum sibiricum* Otnyukova (Pottiaceae), recently described in Siberia [1], seems not only to be distinguished by its morpho-anatomical difference from the rare and threatened *P. kozlovii* Laz. but also by its peculiar ecology. 

Thus, the rare and threatened *P. kozlovii,* whose known range during the last decades has significantly been extended by reports from Asia and North America, seems to be a species complex. The large range is rather the extent of occurrence (EOO) of more than one species, while the real area of occupancy (AOO) remains very small. The AOO for the species within the complex seems to be even smaller with no clear limits or overlapping. However, for such a large EOO, not many recent point records from Europe, Asia, or North America have been made, implying the scattered range and extreme rarity of any of the species from the complex *P. kozlovii*. Consequently, due to its distribution, the gap in knowledge on its ecology, and apparent taxonomic problems, its conservation status remains obscure. 

The tiny moss *Pterygoneurum kozlovii* complex is an extremely rare taxon with limited distribution. It is hard to find in the field due to its small size and seasonal development. Its distribution range is known to be wide but severely fragmented and disjunct in the Northern Hemisphere [2]. It was reported in Europe (Czechia [3]—identity needs reconfirmation—Ukraine [4,5], Slovakia [6], Romania [7], Moldova [8], and Russia—the Rostov and Saratov provinces [8], Tatarstan [9], Bashkortostan, and Kalmykia [10]), Asia (Russian Altai [2], Taimyr [11], and Yakutia [2] as well as Mongolia [12] and China [13]), and North America (restricted to the Canadian territories of British Columbia, Alberta, and Saskatchewan [14]). Pócs [15] suggested this taxon could be found in suitable sites in Hungary. However, it was not reported in recent research in similar habitat types in nearby Croatia and Serbia [16].

No recent target field investigations and reports on the presence of the *Pterygoneurum kozlovii* complex are known, and it is rather hard to find collected specimens in worldwide herbarium collections. Since it has been rarely seen and reported, very limited data on its biology can be found from previous field work and that which are available are mainly in local languages. Thus, novel investigations of this species complex along with a conservation physiology approach to this rare and threatened species/complex are urgently needed, keeping in mind that both the complex and/or the species from it may be lost prior to our learning about its biology and applying appropriate conservation programmes. Establishing in vitro cultures and propagation of such taxa offers not only new insights into species biology but also allows the use of propagated biomaterials for phylogenetic investigations and/or biotechnological approaches. It also decreases the pressure on rare natural populations. 

*Pterygoneurum sibiricum* is faintly distinct from *P. kozlovii.* It can be recognised by the size of the phylloid lamella and the shape of the lamella cell papillae [1]. According to its ecology, the same author assumes and speculates that a strict species of *P. kozlovii* is present only in European countries including the European part of Russia and not in its Asian part, Mongolia, or Canada, where sibling species probably occur. 

It remains unclear if the record reported by Ellis et al. [17] belongs to *P. kozlovii* (in the Asian part of Russia) since the site within the transitional zone from the solonetz steppe to the birch islet is considered, with other salt-tolerant (e.g., *Entosthodon hungaricus* (Boros) Loeske) and weakly salt-tolerant species (e.g., *Physcomitrium arenicola* Laz.), as being reported at the same time.

Thus, the field and empirical knowledge we have on *P. kozlovii* to date is related to the species complex and is rather imprecise. *Pterygoneurum kozlovii* s. str. grows on loamy and sandy terrain, often saline soils in semi-arid steppes to semi-deserts. It is attached to lowlands (reports state 20–200 m altitudes), open grassland sites, and carbonate saline soils [8]. On the contrary, the newly described species (*P. sibiricum*) seems to be present rather on dry mineralised soil, also reaching higher altitudes compared to the dry salty lowland substrate where *P. kozlovii* s. str. can be found. Habitat degradation due to overgrazing, the ploughing of steppes, urbanisation, and trampling can be assumed to be the main threats to this species. Although it can be easily overlooked due to its size and seasonality, there is a continuing decline in habitat quality due to the intensification of land use in its overall distributional range. It is documented that the central European subpopulation is declining and has negative population trends [8], while the population trend in eastern European parts is unknown (no recent reports). 

*Pterygoneurum kozlovii* is regarded as an obligate bryo-halophyte based on the habitat or vegetation types the moss inhabits (e.g., the *Artemisieto-Festucetum pseudovinae* communities in Ukraine, or the grassy margins of alkaline depressions elsewhere) [3,6].

*Pterygoneurum kozlovii* was discovered and first described in the mid-20th century in Ukraine [4]. Almost at the same time, Vanek [18] found and described similar species in former Czechoslovakia and named it *P. smardaeanum* Vanek. Later, Abramova et al. [19] studied type material and synonymised it with the priority name *P. kozlovii.*

Apart from having a peculiar ecology and distribution range, this species is considerably different from other species of the genus *Pterygoneurum.* Some authors consider it of hybrid origin between *Phascum* and *Pterygoneurum* but without providing any clear evidence for this assumption. Thus, Boros [20] regards it as a hybrid of *Phascum cuspidatum* Hedw. (syn. *Tortula acaulon* (With.) R.H. Zander) and *Pterygoneurum ovatum* (Hedw.) Dix, while Corley et al. [21] consider it to be a hybrid between *P. cuspidatum* and *Pterygoneurum subsessile* (Brid.) Jur. The hybrid hypothesis is rather weakly supported bearing in mind the fact that *P. kozlovii* forms normal spores [22]. Other authors assumed its uniqueness to be the result of a specific lifestyle in a carbonated saline environment [23,24]. Boiko [24] even proposed separating it into the monotypic genus *Lazarenkia* (*Lazarenkia kozlovii* Boiko). Here, we follow the nomenclature as considered in Hodgetts and Lockhart [25].

According to Hodgetts et al. [26], based on the assessment of [8], the species is Critically Endangered (CR) both in Europe and the European Union, which according to the areas studied is *P. kozlovii* s. str. It is also regionally and nationally threatened [25]. In Romania [27] and Slovakia [28,29], it is also considered Critically Endangered (IUCN: CR), while in Ukraine it is a rare species (Ukrainian threatened species categorisation, R) [30]. However, in Ukraine (from where it was described), it is known only from two regions: Zaporozh’e [31] and Odesa [23]. In Russia, in the regional red lists, it has been assessed as an endangered species (Russian threatened species categorisation (1b)) in the Rostov province and as a rare species (Russian threatened species categorisation (3)) in the Saratov province [32,33]. The overall population size remains unknown, but in Europe individual equivalents are estimated to be less than 50 [8].

In Canada, *P. kozlovii* is reported to be in decline and linked to threatened ecosystem types. It is not clear if North American populations are related to their European and Asian counterparts or form a separate species, as distinctions in some morphological traits (i.e., spore size) from type material is mentioned [34], but ecological features remain similar to the European accessions, i.e., *P. kozlovii* s. str. The species *Pterygoneurum kozlovii* is protected under federal law in Canada on at least two of its known occurrences: on Department of National Defence lands and National Research Council lands. It is listed as threatened throughout its Canadian range (the provinces of British Columbia, Alberta, and Saskatchewan). This means that all the occurrences and critical habitats of the species which exist on federal public lands have legal protection from destruction, subject to the provisions of the Species at Risk Act [35,36]. It is considered endangered and also stated to be rare in Mongolia [34].

There is no doubt that *P. kozlovii* s. lat. is a rare and obscure species, not only in terms of its biology, but also regarding its conspecificity, ecology, and distribution. Some authors [22] excluded the hybrid origin of *P. kozlovii* s. lat. having in mind its huge but fragmented range; however, with the newly described species from this complex, namely, *P. sibiricum*, hybrid origin as well as hybridisation within the complex, genus, and related taxa are relevant topics to investigate.

Here, we present a study on the propagation and previously unknown biological features of *P. sibiricum,* segregated from the *P. kozlovii* complex, as its taxonomic counterpart, which allows us to also develop techniques which can subsequently be more easily applied to *P. kozlovii* s. str. (see more in [37,38,39]).

Bryophytes have been cultured in axenic conditions and on defined nutritive media since the early 1900s [40]. However, in addition to establishing a contaminant and cohabitant-free bryophyte in vitro system, one of the main problems in bryophyte conservation remains the selection of an appropriate nutrient medium for axenic growth, which is often a difficult and time-consuming task [37,38,39]. The selection of a suitable nutrient medium is an important step for each developmental stage of bryophytes, such as spore germination, the development of vegetative propagules, and promoting gametophores and protonemal growth [41,42]. Many developmental, physiological, and metabolic processes in bryophytes are at least partly regulated by nutrient availability [43]. Thus, it is of great importance to investigate the effects of basal nutrient media and exogenously added sugars on the developmental processes of different bryophyte species so as to discover their functions in this group of plants.

We apply a conservation physiology approach to learn about obscure species biology and to speed up the acquisition of knowledge relating to rare and threatened species. Features such as plant nutrition, reaction to growth hormones, sugars, or even culture conditions may be relevant to understanding the biology and ecology of the target species, i.e., reactions to the environment, spore germination, dormancy, ecological preferentials, developmental optimums, and receiving and transferring signals. These cannot be easily achieved in rare, scattered, and small native populations. Furthermore, the species complex can be hard to distinguish, and physiological laboratory tests can confirm or reject the taxonomic position of the strict species from the complex by providing a specific ecological niche and thus implying its good or bad taxonomical position. Some of the species from the complex cannot be distinguished in the field; thus, laboratory tests offer more valuable data.

The aims of this study were as follows: (1) establish an axenic in vitro culture of *P. sibiricum*; (2) define the conditions needed for the propagation and ex situ conservation of *P. sibiricum* gametophytes and their easy micropropagation; (3) investigate the effects of the essential growth regulators auxin and cytokinin, as well as selected sugars on the growth, development, and biomass production of this moss species; and (4) examine its salt tolerance since it was not clear whether *P. sibiricum* was able to cope with increased salinity, as reported for its taxonomic counterpart *Pterygoneurum kozlovii* which effectively survives salt stress conditions.

## 2. Results

### 2.1. The Influence of Nutrient Medium and Exogenously Added Sugars on Pterygoneurum sibiricum morphogenesis

**Figure 1:**
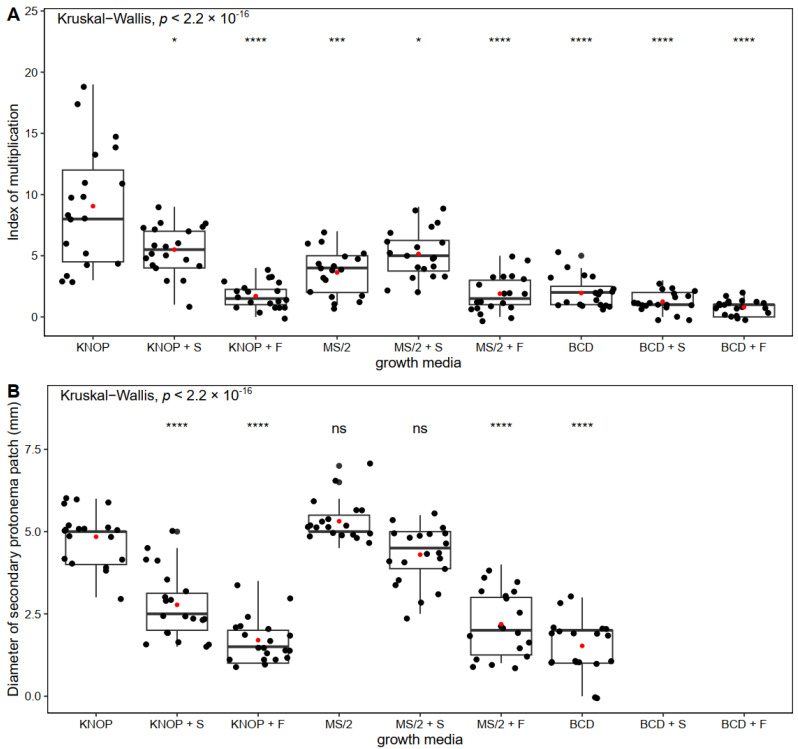
The index of multiplication (**A**) and secondary protonemal patch diameter (**B**) of *Pterygoneurum sibiricum* on different nutrient media in Experiment type I. The box ranges from the first (Q1) to the third (Q3) quartile, with the black horizontal line representing the median. The black dots represent individual observations, while the red dots represent the mean. The whiskers extend to the 1.5 × IQR (interquartile range) from the edge of the box. Comparisons were made with the KNOP growth media as the reference group using the Wilcoxon rank-sum test * *p* ≤ 0.05; *** *p* ≤ 0.001; **** *p* ≤ 0.0001; ns—nonsignificant.

**Figure 2:**
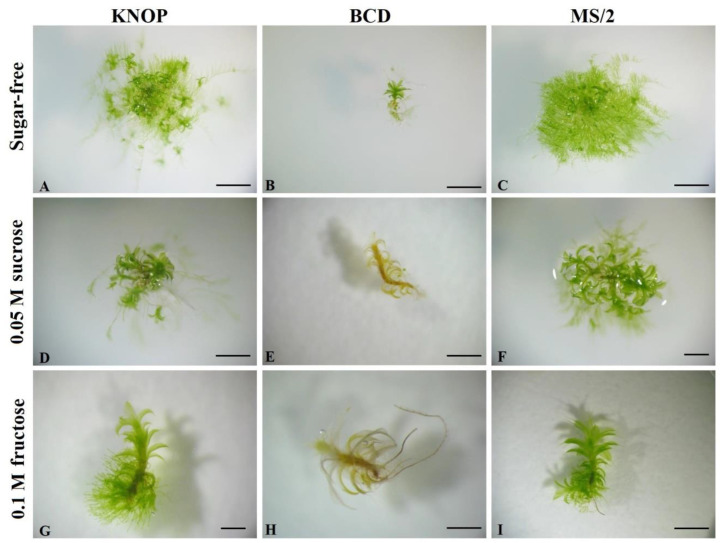
The appearance of *Pterygoneurum sibiricum* explants grown on different sugar-free nutrient media (**A**–**C**) and supplemented with sucrose (**D**–**F**) and fructose (**G**–**I**) in Experiment type (**I**). The bars represent size ((**A**–**E**,**H**,**I**) 4 mm; (**F**,**G**) 2 mm).

In Experiment type I, *P. sibiricum* formed the greatest number of new gametophores when grown on the basal KNOP medium compared to all the other tested growth media (Figure 1A). The plants developed normally, i.e., green gametophores were present, and secondary protonemal patches were observed when grown on a nutrient sugar-free substrate (Figure 2A–C). Exogenously added sugars (sucrose and fructose) express an inhibitory effect on the formation of new shoots and buds (Figure 1A). Sugars added to the BCD medium led to a lethal outcome for the plantlets (Figure 2E,H). Nevertheless, when the plants were grown on the KNOP and MS/2 media supplemented with 0.05 M sucrose, new shoots were documented but to a lesser extent compared to the KNOP sugar-free nutrient media. On the other hand, the plants grown on the BCD medium enriched with sucrose (BCD + S) or fructose (BCD + F) did not survive, i.e., no index of multiplication was recorded (IM). Thus, any newly formed plantlets were documented (Figure 1A) because of the sublethal conditions.

Nevertheless, the largest diameter of the secondary protonema patch was measured in the plants grown on the MS/2 nutrient medium (Figure 1B) although protonemata were also present and measurable in the plants grown on the KNOP basal media and MS/2 supplemented with sucrose (MS/2 + S) (Figure 2B). However, no statistically significant differences were recorded between the plants grown on the KNOP and MS/2 media or between the KNOP and MS/2 + S growth media related to the diameters of the secondary protonema patches (Figure 1B), which suggests the possible suitability of those media for in vitro protonemal growth and moss development. In general, the addition of sucrose to the nutrient media was more effective than fructose for the development of *P. sibiricum* under in vitro morphogenesis.

In addition to the IM and secondary protonemal patch diameter, morphogenetic appearance was also examined (Figure 2) in order to define the most suitable nutrient media for the micropropagation of *P. sibiricum*. Overall, based on the comparison of all the obtained results in Experiment type I, the KNOP and MS/2 nutrient media proved to be suitable for the growth and micropropagation of *P. sibiricum* in vitro. However, for the purpose of growing more protonema than shoots, MS/2 supplemented with sucrose may also be used, while the BCD nutrient medium was generally inadequate for the development of this species. Moreover, *P. sibiricum* does not require sucrose or fructose for better growth, although sucrose may promote the formation of secondary protonema patches when combined with MS/2 nutrient media.

### 2.2. The Influence of Growth Regulators on Pterygoneurum sibiricum morphogenesis

In order to examine the morpho-anatomical and physiological reactions of *P. sibiricum* on exogenous growth regulators, the plants were grown on a KNOP nutrient medium supplemented with different IBA and BAP concentrations. KNOP basal medium was chosen as a good solution based on the results obtained in Experiment type I.

**Figure 3:**
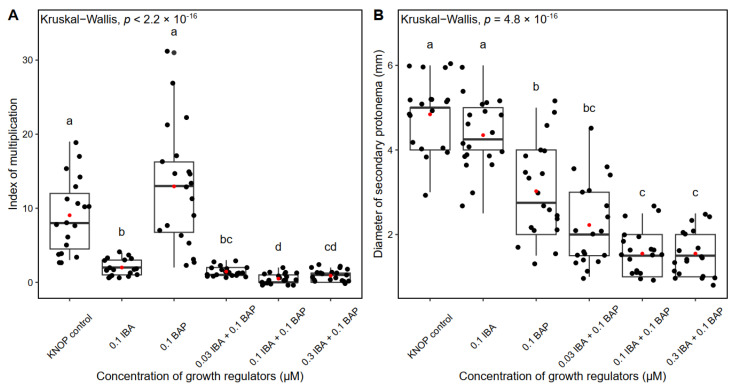
The index of multiplication (**A**) and secondary protonemal patch diameter (**B**) of *Pterygoneurum sibiricum* on the KNOP nutrient media supplemented with growth regulators in Experiment type II. The box ranges from the first (Q1) to the third (Q3) quartile, with the black horizontal line representing the median. The black dots represent individual observations, while the red dots represent the mean. The whiskers extend to the 1.5 × IQR (interquartile range) from the edge of the box. The letters above the boxplots indicate the statistically significant differences among the experimental groups (*p* < 0.05 after Dunn’s multiple comparisons test).

The highest number of new shoots and buds were documented in those plants grown on the KNOP nutrient medium supplemented with 0.1 µM BAP, which was similar to that observed in the control group of plants (Figure 3A). However, no statistically significant differences were documented between these two groups, suggesting that the control group plants and those grown on 0.1 µM BAP developed a similar number of new shoots (Figure 3A). In general, the growth regulators inhibited the formation of new shoots, whether applied individually or combined. Thus, the IM was significantly lower and fairly uniform compared to the control plants (Figure 3A). Statistically significant differences were observed between the control group and the other experimental groups (*p* < 0.001), with the exception of 0.1 µm BAP which also showed significant differences compared to all the other experimental groups except for the control group (*p* < 0.001).

The largest secondary protonemal diameter was documented for the control group plants and the plants grown on the KNOP nutrient medium supplemented with 0.1 µM IBA (Figure 3B). Increased concentrations of combined IBA and BAP (0.1 µM IBA + 0.1 µM BAP, and 0.3 µM IBA + 0.1 µM BAP) led to decreasing protonemal patch diameters, suggesting that the growth regulators had no positive effect on *P. sibiricum* morphogenesis (Figure 3B). Statistically significant differences were observed between the control group and the other experimental groups (*p* < 0.01), with the exception of 0.1 µm BAP which also showed significant differences compared to all the other experimental groups except for the control group (*p* < 0.05).

**Figure 4:**
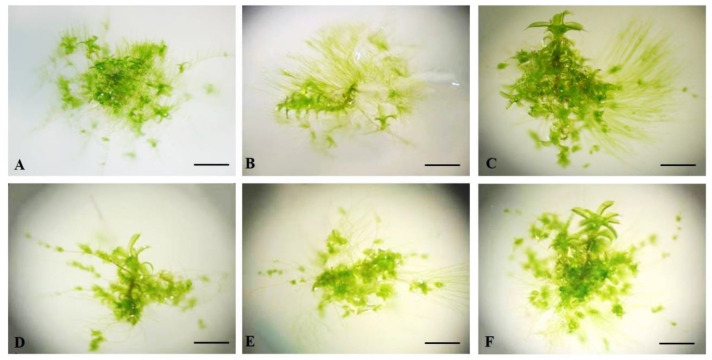
The appearance of *Pterygoneurum sibiricum* explants grown on KNOP nutrient medium supplemented with IBA and BAP in Experiment type II: (**A**) PGR-free; (**B**) 0.1 µM IBA; (**C**) 0.1 µM BAP; (**D**) 0.03 µM IBA + 0.1 µM BAP; (**E**) 0.1 µM IBA + 0.1 µM BAP; (**F**) 0.3 µM IBA + 0.1 µM BAP. The bars represent size ((**A**–**F**) 4 mm).

The plants grown on the KNOP nutrient medium supplemented with different combinations of growth regulators IBA and BAP developed normally, i.e., green gametophores and secondary protonema patches were present (Figure 4).

Moreover, the tested growth regulators led to morpho-anatomical changes, indicating that *P. sibiricum* is sensitive to exogenous IBA and BAP and that one of the developmental strategies of the examined species is to form many bulbil-like protonemal buds for new gametophores and to further moss patch development (Figure 4C–F). In general, exogenously added selected plant growth regulators are not necessary for the propagation of *P. sibiricum*, having no strong effect on mass production. However, they can be useful agents for the purpose of protonemal bud induction.

### 2.3. The Influence of NaCl on Pterygoneurum sibiricum morphogenesis

**Figure 5:**
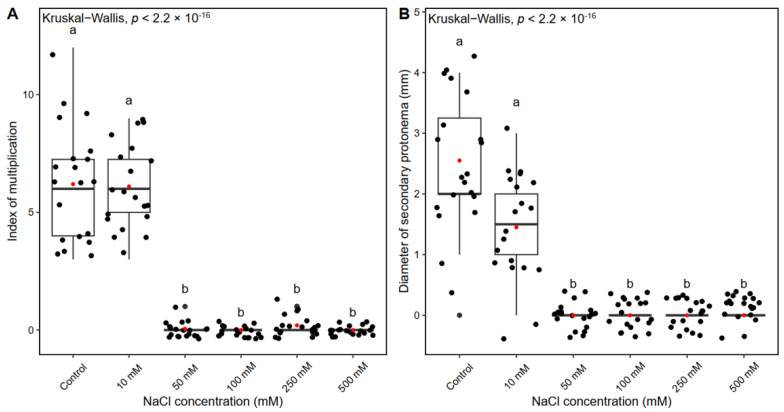
The index of multiplication (**A**) and secondary protonemal patch diameter (**B**) of *Pterygoneurum sibiricum* on KNOP nutrient media supplemented with different NaCl concentrations in Experiment type III. The box ranges from the first (Q1) to the third (Q3) quartile, with the black horizontal line representing the median. The black dots represent individual observations, while the red dots represent the mean. The whiskers extend to the 1.5 × IQR (interquartile range) from the edge of the box. The letters above the boxplots indicate statistically significant differences among the experimental groups (*p* < 0.05 after Dunn’s multiple comparisons test).

**Figure 6:**
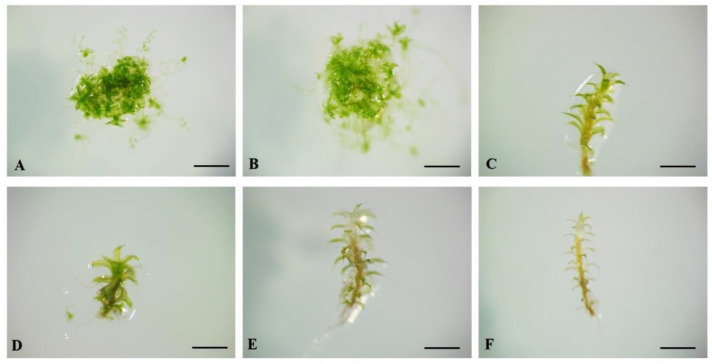
The appearance of *Pterygoneurum sibiricum* explants grown on KNOP nutrient medium supplemented with different NaCl concentrations: (**A**) control; (**B**) 10 mM NaCl; (**C**) 50 mM NaCl; (**D**) 100 mM NaCl; (**E**) 250 mM NaCl; (**F**) 500 mM NaCl. The bars represent size ((**A**–**F**) 4 mm).

According to the recent morpho-anatomical segregation of this species outside salt-tolerant species *P. kozlovii*, and due to its habitat preferentials, it is also expected to be ecologically segregated from *P. kozlovii* s. str. In order to examine the physiological reactions of *P. sibiricum* to salt stress, the plants were grown on a KNOP nutritional medium supplemented with different NaCl concentrations (Table 1). In general, in *P. sibiricum,* the IM decreased with the addition of increasing concentrations of NaCl to the nutrient medium (Figure 5A). All the experimental groups showed a significantly lower number of newly formed shoots compared to the control group plants (*p* < 0.0001) and 10 mM NaCl-treated plants (*p* < 0.0001) (Figure 5A). The plants developed normally when grown on the KNOP nutrient medium and nutrient medium supplemented with a low concentration of NaCl, i.e., green gametophores and secondary protonema patches were documented (Figure 6A,B). Therefore, a low concentration of NaCl did not affect the development of new shoots in *P. sibiricum,* suggesting some tolerance to NaCl, since the IM was similar to that in the control group plants (Figure 5A). However, increased concentrations of NaCl (50–500 mM) negatively affected the formation of new shoots and secondary protonema patches (Figure 5A,B), suggesting survival in a suboptimal salt environment. Moreover, signs of chlorosis and loss of pigment were noticeable in the plants when NaCl was added to the medium (Figure 6C–F). In addition, extremely high NaCl concentration (500 mM) had a lethal effect on *P. sibiricum*, i.e., no plants survived such conditions (Figure 6F).

Increased concentrations of NaCl (50–500 mM) inhibited the formation of secondary protonemal patches as inferred by the patch diameter (Figure 5B). All the experimental groups showed significantly lower protonemal patch diameters compared to the control plants (*p* < 0.0001) and 10 mM NaCl-treated plants (*p* < 0.0001), with no significance shown between these two groups (the control group plants and 10 mM NaCl-treated plants).

**Figure 7:**
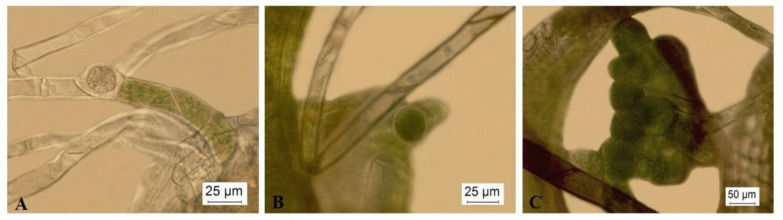
The formation of brachycytes in *Pterygoneurum sibiricum* explants grown on KNOP nutrient medium supplemented with different NaCl concentrations: (**A**) 50 mM NaCl; (**B**) 100 mM NaCl; (**C**) 250 mM NaCl.

Increased NaCl concentrations also induced morpho-anatomical changes in *P. sibiricum*. Moderate (50–100 mM) and rather extreme NaCl concentrations (250 mM) induced the formation of “brood” cells or brachycytes (Figure 7). Brachycytes serve as vegetative diaspores for the survival of adverse environmental conditions. Moreover, a greater number of brachycytes were observed in those plants grown on the KNOP nutrient medium supplemented with 250 mM (Figure 7C) than those grown on the KNOP nutrient medium supplemented with 50 and 100 mM (Figure 7A,B). According to those results, it can be assumed that under salt stress conditions, the surviving plants used their resources and energy for their survival and potential defence mechanisms as despite the fact that these plants can survive moderate concentrations of NaCl, under stressed conditions, *P. sibiricum* did not develop new shoots. Thus, this species seems not to be a bryo-halophyte and grows better on substrates without salt, although it can tolerate a small amount of salt during the peak of its vegetative season, i.e., a short period of other positive environmental factors (i.e., full moss hydration).

## 3. Discussion

### 3.1. The Influence of Nutrient Medium and Exogenously Added Sugars on Pterygoneurum Sibiricum Morphogenesis

In this study, three different nutrient media were used for propagation and development studies of *P. sibiricum*. According to the results obtained in Experiment type I, the highest number of newly and normally formed shoots were recorded in the plants grown on the KNOP nutrient medium. However, the secondary protonemal patch developed better in those plants grown on the MS/2 medium (Figure 1B), suggesting that both KNOP and MS/2 are equally adequate for plant conservation and can be applied if vegetative parts are needed (gametophores vs. protonemal threads). On the other hand, the BCD medium exerted harmful effects on *P. sibiricum* as assumed by its growth on these media types, especially when combined with sucrose or fructose (Figure 1 and Figure 2). The reason for such results is the composition of these media since all the other growth conditions were equal during the tests. One of the constituents of BCD is nitrate salt (in the form of KNO_3_), which is a possible culprit for the unusual developmental behaviour of *P. sibiricum*. This is not surprising since *P. sibiricum* is not found in nitrificated but rather nutrient-poor microhabitats. Thus, the presence of nitrate in the substrate can be a limiting factor for the settlement, spread, or even spore germination of this species. On the contrary, KNOP and MS/2 nutrient media are richer in mineral salt compared to BCD, which is in accordance with the poor mineral substrate where this species is found in nature.

Previous research has also shown that certain types of nutrient media are more suitable for the propagation of mosses in vitro and that some species often prefer solid nutrient media free of sugars or growth regulators [39,44,45,46]. However, low concentrations of exogenously added sucrose or NaCl to BCD or KNOP media for shorter periods can contribute to the improved multiplication and regeneration of some tested species, such as bryo-halophyte *Hennediella heimii* (Hedw.) R. H. Zander [46,47,48]. In general, sugars such as sucrose, fructose, and glucose have essential functions in plant metabolism [49] but do not necessarily contribute to the increase in IM and improved growth of mosses [45] since bryophytes are rather successful in autotrophic culture systems compared to vascular plants (photomixotrophic metabolism in culture) [50]. There is very diverse data on the influence of sugar on the formation of new shoots and buds in mosses in vitro in the existing literature. The effects of sugar on bryophytes are rather poorly documented, and its use in the cultivation of tracheophytes cannot be extrapolated easily as sugars can act as a carbon source, signal molecule, or both. In this study, the results undoubtedly showed that the addition of sucrose or fructose was inadequate for *P. sibiricum* propagation. Similar results were obtained for the moss *Atrichum undulatum* (Hedw.) P. Beauv., which formed only a few newly developed shoots when grown on nutrient media enriched with sugars but was fully developed when grown on sugar-free media [43]. On the other hand, the formation of new buds was promoted by the addition of sucrose and glucose to the nutrient medium in *Pohlia nutans* (Hedw.) Lindb. [51] and *Funaria hygrometrica* Hedw. [52]. Additionally, in mosses *Leptobryum pyriforme* (Hedw.) Wilson and *Barbula gregaria* (Mitt.) A. Jaeger, new buds were completely absent in plants grown in nutrient media free of sucrose [51]. Some other species formed more new thalli when grown on nutrient media supplemented with sucrose and fructose, such as the liverwort *Riccia crystallina* L. [52]. Moreover, the formation of secondary protonema patches was promoted in *Bryum argenteum* Hedw. as well as sex organ induction by fructose added in optimal concentrations to nutrient media (0.01–0.05 M) [43]. On the contrary, sucrose (30 g/L, i.e., 1M) had little to no effect on increased bud formation and secondary protonema diameter in *Splachnum ampullaceum* Hedw. for long-term cultivation on ammonium nutrient medium [53]. The existence of such contradictory results in the literature for examined bryophytes indicates species-specific responses and various developmental strategies which occur in bryophytes that are often in accordance with their mineral requirements. Various sugar types, their conjugates, and their concentration applied in various laboratory conditions make the puzzle of sugar effects even more blurred, having in mind that they can have overlapping and finely regulated constitutional, signalling, or energy supplier roles in bryophytes. The obtained results on the effects of sugar on *P. sibiricum* are not unexpected, but further investigation implying a wider spectrum of sugar types and their amounts under different growth conditions is needed to gain a deeper understanding of these phenomena.

### 3.2. The Influence of Growth Regulators on Pterygoneurum sibiricum morphogenesis

Some moss species spontaneously form a large number of new shoots in axenic conditions, such as model moss *Physcomitrium patens* (Hedw.) Mitt., while others need the presence of growth regulators or sugars in the nutrient media or some other stimuli for bud induction, e.g., [46,54,55]. Previous studies have described the effects of auxin and cytokinin on morphogenesis in bryophytes. However, there is still a lack of data on their combined influence, i.e., the synergistic or antagonistic effects [38,45].

In general, it is clear from studies [56] that bryophytes respond to exogenously added growth regulators and exhibit certain developmental responses. Moreover, mosses synthesise cytokinins and auxins in specific forms, mostly in the cisZ type in nature, the iP type (N6-(Δ2-isopentenyl) adenine) in controlled conditions, and free indol-3-acetic acid (IAA), respectively [57]. Nevertheless, there are still insufficient data on the optimal concentrations of growth regulators for the initiation of certain developmental processes in bryophytes. Therefore, more studies are needed to discover the possible and general patterns of the effects of growth regulators on morphogenesis in order to use that information for better conservation and micropropagation of bryophytes.

In this study, the external addition of IBA and BAP in combination had an inhibitory effect on new shoots and bud formation in *P. sibiricum*. However, when applied individually, 0.1 µM BAP promoted the formation of new shoots, which was not completely unexpected since cytokinins are known to play a pivotal role in bud formation and also due to their localisation on the caulonema [58]. In low concentrations, cytokinins lead to the normal development of gametophores in mosses as was shown for *A. undulatum* when BAP was applied [59]. In contrast, high concentrations of cytokinins often induce the development of abnormal gametophores and a small number of new buds and shoots [52]. This was also proven for *H. heimii* when 10 µM BAP was used [46], as well as for *B. argenteum* [59]. Moreover, in this study, when combined with IBA, concentrations of BAP higher than 0.1 µM had negative effects on the formation of new shoots (Figure 3A). Cytokinins mainly affect bud formation on caulonemal cells in formed protonema [60].

Chopra and Kumra [52] stated that the age and size of protonemal patches are species-dependent and are crucial for the beginning of bud formation, implying the level of endogenously produced growth regulators. However, the addition of combined IBA and BAP to growth media induced the formation of a smaller secondary protonemal patch in *P. sibiricum* (Figure 3B) in comparison with the control group plants. Nevertheless, mosses grown on nutrient media supplemented with 0.1 µM IBA developed a similar secondary protonemal patch diameter to the control plants. The combination of IBA and BAP negatively affected the formation of protonema, and the caulonema was dominant in relation to the chloronemal threads. This pattern was previously observed in *Hypnum cupressiforme* Hedw. [45], where the caulonema developed better than the chloronema under similar treatments. Moreover, higher concentrations of IBA and BAP led to a reduction in the protonemal patch diameter, suggesting that high concentrations of growth regulators demonstrated inhibitory effects on protonemal patch diameter. A similar pattern was previously documented for *H. heimii* [46] and *B. argenteum* [59], where the critical concentration of IBA was 0.1 µM. The plants grown on media supplemented with low concentrations of BAP (0.1 µM) developed slightly smaller protonemal patches than the control plants and those grown on media supplemented with 0.1 µM IBA (Figure 3B). The opposite results were obtained in *H. heimiii,* where low concentrations of BAP (0.03 and 0.1 µM) had a positive impact on protonemal patch growth. Thus, previous results clearly indicate that cytokinins are required for a certain period of time to induce caulonemal differentiation and growth as well as the production of buds in bryophytes, see also [61,62]. According to the present knowledge, IBA can enhance protonemal growth in an optimal concentration range. However, very low and high concentrations are inhibitory for protonemal development in axenic conditions [63], and the range and exposure time required seem to vary between species. Thus, IBA should be applied for shorter periods of time in order to promote bud formation since high concentrations and long exposure to IBA had negative outcomes in this experiment. Moreover, the prolonged application of growth regulators often had a negative effect on moss morphogenesis, as was previously shown for *Bryum capillare* Hedw. [64]. Therefore, auxins and cytokinins must be applied in optimal concentrations and for optimal durations for individual species in order to induce and promote moss growth and development.

In general, a low level of auxins seems to be the key factor for protonemal differentiation and bud formation, i.e., the development of the bud primordia, whereas high concentrations of auxin are responsible for gametophore development [65]. It was previously shown that auxins, such as IBA, control the transition of chloronema to caulonema [66,67] and promote the formation of rhizoids and vegetative gemmae [68] in optimal concentrations; thus, the obtained results for *P. sibiricum* were to be expected.

### 3.3. The Influence of NaCl on Pterygoneurum sibiricum morphogenesis

Bryophytes are generally considered not to be halophytes, although some species can effectively tolerate high salinity by engaging developmental and/or biochemical stress tolerance mechanisms [44,69]. During exposure to NaCl, the initial reaction of plants is the inhibition of cell expansion and division [70,71]. Bryophytes can react to increased habitat salinity in many ways, depending on their sensitivity to salt stress. Generally, all the tested parameters of morphogenesis decrease with the increase in salt concentrations, as was documented in previous studies [47,48]. Survival and the formation of secondary protonema and new shoots decline rapidly in salt-sensitive species after short- or long-term exposure to salt stress in axenic conditions, as shown for *A. undulatum* [72]. Moreover, different species such as *B. argenteum* responded to NaCl with a decline in survival and a reduction in the number of new shoots and buds [72]. Similar results have been observed in salt-tolerant species, and such a decline in survival and decrease in the production of new shoots and secondary protonema occur in these species after long-term exposure to NaCl [47,48].

In this research, the plants grown on the nutrient medium supplemented with 500 mM NaCl did not survive at all, whereas other plants survived even high concentrations of NaCl (250 mM), although their appearance was rather different than that of the plants in the control group (Figure 6F). Knowing that *P. sibiricum* is recorded in a non-halophytic environment, it was good to document its survival at a rather high NaCl concentration (250 mM). This allows us to infer that there is a segregation of *P. sibiricum* from *P. kozlovii* s. str., it was a recent event, and although *P. sibiricum* ecologically prefers non-salt environments, it can survive and support such habitat types for short periods of time, especially if the salt effect is antagonised by other environmental factors. This also serves to explain the reason for some *P. kozlovii* s. lat. incl. *P. sibiricum* reports from ecotones of harsh salty environments to non-salty habitats (e.g., the transitional zone from the solonetz steppe to birch islet, [17]). Special attention should be paid when specimens are found to grow in such peculiar ecological conditions, and both species seem to be plastic enough to appear in such places sympatrically.

Nevertheless, the plants of *P. sibiricum* grown on media enriched with 50–250 mM NaCl developed brachycytes (Figure 7), suggesting suboptimal growth conditions. Such “brood cells” or gemma-like structures are rounded, thick-walled cells developed in the protonema which serve as vegetative diaspores for surviving harsh environmental conditions or poor vegetative seasons [73,74,75,76]. The presence of brachycytes in the protonema of *P. sibiricum* increases its ability to survive for short periods in highly salty environments.

Although some of the bryophyte species tested to date were not empirically classified as bryo-halophytes, they were able to survive very high concentrations of NaCl added to the nutrient media, (e.g., *P. patens*; [77]). Moreover, *P. patens* survived better on extremely high concentrations of NaCl than some ecologically obligate bryo-halophytes such as *H. heimii* and *E. hungaricus* [47,48], showing rather good salt tolerance. Indeed, Frank et al. [77] studied the relationship of *P. patens*’s tolerance to drought, osmotic stress, and salt stress. Furthermore, it was documented that the survival rate increased in salt-sensitive species if the exposure to salts was gradual, even up to 600 mM [78].

On the other hand, low concentrations can stimulate the formation of new shoots and secondary protonema, especially in bryo-halophytes such as *H. heimii* and *E. hugnaricus* [47]. In this study, the formation of new shoots in *P. sibiricum* was already sensitive at lower NaCl concentrations (10 mM NaCl), while for the same treatment secondary protonema developed similarly to control group plants, suggesting that low concentrations of NaCl were not harmful to protonema development. In general, pottioid mosses tend to allocate energy to protonemal growth in salt stress conditions, compared to funarioid mosses which form more shoots than protonema [47], which is also confirmed for pottioid *P. sibiricum* in this study.

## 4. Materials and Methods

### 4.1. Plant Material

The specimen of the *P. kozlovii* complex was collected in the Kosh-Agach District on the Yuzhno-Chuysky Range (Russia) in the dry steppes on steep slopes on the side of the Tarkhata River valley, 2240 m a.s.l., 49°37′44″ N, 88°27′13″ E, 21 June 2021, leg. Ignatov M.S. & Ignatova E.A. (#21-573). The voucher specimen is kept in the collection of the Main Botanical Garden of the Russian Academy of Sciences, MHA9130928. Re-examination of the material shows this accession fits in *P. sibiricum.*

Two dry sporophytes (mature capsules) from the aforementioned MHA9130928 sample were used as the starting material for the establishment of axenic in vitro cultures as previously elaborated, e.g., [41,45,54,79,80].

Capsules of the herbarium material were separated, cleansed of mechanical impurities, and carefully washed with distilled water to avoid spore loss. They were immersed in sodium hypochlorite (NaOCl) solution of various concentrations (1%, 3%, 5%, 7%, 10%, and 13%) with the aim of finding one that would kill all the remaining germs and contaminating cohabitants and at the same time circumvent target moss spore damage. Additionally, during the sterilisation process, the exposure time to each solution was also varied (duration 60, 90, 120, and 240 s) in order to achieve non-contaminated spores prior to spreading them on selected media types under the flow chamber.

After the sterilisation process, the spore germinability as well as contaminant organism remnants were tested on the KNOP basal medium (for the KNOP medium content, see [81,82]). The plant material was grown in sterile chambers at a constant temperature (18 ± 2 °C), humidity 60–70%, and a long-day light regime (16 h light/8 h dark) for eight weeks and was constantly checked for infections. Fluorescent tubes (Tesla Pancevo) were used as the light source with a flux density of 50 µmol m^−2^ s^−1^.

### 4.2. In Vitro Micropropagation of the Plant Material

After the establishment of axenic cultures, i.e., the achievement of axenic plantlets, the plants were propagated on a minimal KNOP nutrient medium [81] until the gametophores reached the optimal size for experimentation. The problems and growth optimisation of in vitro bryophytes are elaborated elsewhere, e.g., [37,38,39]. For further examination, each explant was placed in Petri dishes with the appropriate type of medium. The pH of the media was adjusted to 5.8 prior to autoclaving at 121 °C for 45 min. A single gametophore 5 mm in length was used as the initial explant. There were five replications of each treatment (Petri dishes), each containing 10 gametophores. The experiment was performed in axenic conditions free from uncontrolled cohabitant effects or unpredicted abiotic condition variations.

### 4.3. Experimental Design

In this research, several types of experiments were performed.

In Experiment type I, the effects of nutrient media and exogenously added sugars on the target moss morphogenesis were examined. The explants were grown on minimal KNOP medium [81], MS medium at half strength (referred to as MS/2) [83], and BCD medium [84] subsequently enriched with 15% sucrose (0.05 M) or fructose (0.1 M). The sugar concentrations were chosen so as to apply sucrose at half strength, as is commonly used in vascular plant tissue culture (the photomixotrophic vs. autotrophic system in bryophytes). Sucrose converts rather quickly into equimolar amounts of glucose and fructose, and the glucose is then taken up preferentially into the plant cells, e.g., [85]. Fructose is documented to have a lower affinity by plant membrane hexose carriers (up to eightfold); thus, we chose 0.1 M fructose (twofold concentration of sucrose) to test its effects in mosses.

In order to examine the effects of exogenous plant growth regulators (PGR), the plants were grown on minimal KNOP medium supplemented with various concentrations of indole-3-butyric acid (IBA) and 6-benzylamino purine (BAP) both individually and combined (Experiment type II). The concentrations of IBA and BAP used in these experiments are given in Table 1. 

In Experiment type III, the effect of NaCl was studied. The plants were grown on a minimal KNOP medium enriched with various concentrations of NaCl (Table 1).

After 4 weeks of experimentation, the morphogenetic changes (index of multiplication (IM) and secondary protonemal patch diameter) were measured and documented using a Leica MZ stereo microscope (Leica MZ 7.5 Bi-Optic Inc. Santa Clara, CA, USA) and a conventional light microscope (Leica DMLS, Santa Clara, CA, USA). The index of multiplication (IM) represents the newly formed shoots which originated from the newly induced buds on the secondary protonema patch developed from the initial explant [47,86].

### 4.4. Statistical Analysis

Statistical analysis was carried out using R programming language (v. 4.3.1) [87]. The data were assessed using the Shapiro–Wilk normality test and Lavene’s test of homogeneity of variance, where it was shown that not all the experimental groups were normally distributed, and the homogeneity of variance assumption was violated across the groups. Thus, a non-parametric test, the Kruskal–Wallis test, was used for a comparison of the experimental groups. Afterward, two different post hoc tests were used. The Wilcoxon rank-sum test was used for the comparisons to the reference group in Experiment type I, and Dunn’s multiple comparisons test with the Benjamini–Hochberg *p*-value adjustment method was used for multiple comparisons between the groups in Experiments type II and III. *p*-values lower than the significance level (α) of 0.05 were considered statistically significant.

## 5. Conclusions

The peculiar moss *P. sibiricum,* recently segregated from the *P. kozlovii* complex, has rather different biological characteristics. The species is rare and tiny and the conservation physiology approach allows us to understand that although the species is able to survive in rather salty environmental conditions for short periods, it avoids such habitats in contrast to *P. kozlovii* s.str. *Pterygoneurum sibiricum* is affected by exogenously applied plant growth regulators which can be used in the micropropagation of this interesting and rare species. The results obtained also suggest the possibility of the two sibling species overlapping in peculiar ecological situations where they can thrive sympatrically. The ex situ collection, achieved during this investigation, provides not only the conservation background for this species but also further investigation of the hybridisation both within and outside this species complex, as well as other studies ranging from basic phylogenetics to phytochemical analyses.

## Figures and Tables

**Table 1:** Experimental design summary. Each treatment included 20 replicates. The growth conditions were as described in Section 4.1.

Experiment type I	KNOP (control): KNOP minimal medium, sugar-free MS/2 (control): MS mineral salts, half strength, sugar-free BCD (control): BCD mineral salts, sugar-free KNOP + S: KNOP minimal medium enriched with 0.05 M sucrose KNOP + F: KNOP minimal medium enriched with 0.1 M fructose MS/2 + S: MS mineral salts, half strength, enriched with 0.05 M sucrose MS/2 + F: MS mineral salts, half strength, enriched with 0.1 M fructose BCD + S: BCD mineral salts enriched with 0.05 M sucrose BCD + F: BCD mineral salts enriched with 0.1 M fructose
Experiment type II	KNOP (control): KNOP minimal medium, PGR free KNOP supplemented with 0.1 µm IBA KNOP supplemented with 0.1 µm BAP KNOP supplemented with 0.03 µm IBA and 0.1 µm BAP KNOP supplemented with 0.1 µm IBA and 0.1 µm BAP KNOP supplemented with 0.3 µm IBA and 0.1 µm BAP
Experiment type III	KNOP (control): KNOP minimal medium, NaCl free KNOP supplemented with 10 mM NaCl KNOP supplemented with 50 mM NaCl KNOP supplemented with 100 mM NaCl KNOP supplemented with 250 mM NaCl KNOP supplemented with 500 mM NaCl

## Data Availability

All the data are available by authors upon request.
